# Nanoscale Control of DNA-Linked MoS_2_-Quantum Dot Heterostructures

**DOI:** 10.1021/acs.bioconjchem.2c00285

**Published:** 2022-08-15

**Authors:** Teymour Talha-Dean, Kai Chen, Giulia Mastroianni, Felice Gesuele, Jan Mol, Matteo Palma

**Affiliations:** † Department of Physics and Astronomy, Queen Mary University of London, London, E1 4NS, United Kingdom; ‡ Department of Chemistry, Queen Mary University of London, London, E1 4NS, United Kingdom; § Institute of Materials Research and Engineering (IMRE), Agency for Science, Technology and Research (A*STAR), 138634, Singapore; ⊥ School of Biological and Behavioral Sciences, Queen Mary University of London, London, E1 4NS, United Kingdom; ∥ Department of Physics “Ettore Pancini”, University of Naples “Federico II”, Via Cinthia, 21 Ed. 6, 80126 Napoli, Italy

## Abstract

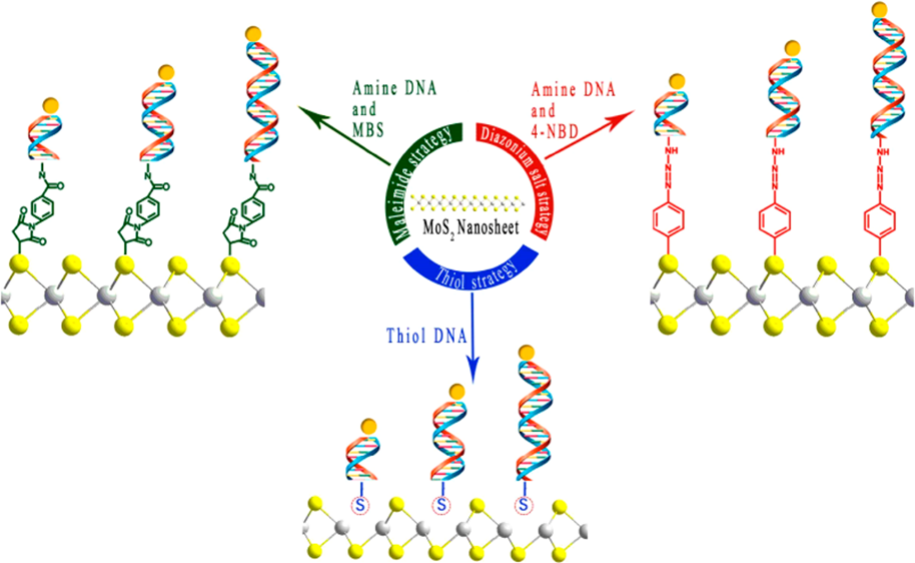

The ability to control the assembly of mixed-dimensional heterostructures with nanoscale control is key for the fabrication of novel nanohybrid systems with new functionalities, particularly for optoelectronics applications. Herein we report a strategy to control the assembly of heterostructures and tune their electronic coupling employing DNA as a linker. We functionalized MoS_2_ nanosheets (NSs) with biotin-terminated dsDNA employing three different chemical strategies, namely, thiol, maleimide, and aryl diazonium. This allowed us to then tether streptavidinated quantum dots (QDs) to the DNA functionalized MoS_2_ surface via biotin–avidin recognition. Nanoscale control over the separation between QDs and NSs was achieved by varying the number of base pairs (bp) constituting the DNA linker, between 10, 20, and 30 bp, corresponding to separations of 3.4, 6.8, and 13.6 nm, respectively. Spectroscopic data confirmed the successful functionalization, while atomic force and transmission electron microscopy were employed to image the nanohybrids. In solution steady-state and time-resolved photoluminescence demonstrated the electronic coupling between the two nanostructures, that in turn was observed to progressively scale as a function of DNA linker employed and hence distance between the two nanomoieties in the hybrids.

Integrating nanoscale materials with different properties can lead to the formation of novel nanohybrid systems with new functionalities, particularly for optoelectronics applications.^1−6^ In this regard, low dimensional materials have attracted much scientific interest in recent years due to the tuning of their properties as a function of size.^7^ Nanomaterials exhibiting a sub-100 nm size in all three spatial dimensions are known as zero-dimensional (0D) materials; an example of such a structure is found in semiconducting nanocrystals (quantum dots, QDs),^8^ resulting in a high degree of electronic confinement. Confinement in only one spatial direction yields low dimensional materials in 2D planar sheet form, known as nanosheets (NSs).^9^ Combining various low dimensional materials into functional heterostructures allows for the exploitation of their nanoscale properties, potentially resulting in novel interactions and applications.^2,10,11^

MoS_2_, a layered van der Waals material, exhibits weak electrostatic interlayer forces and strong covalent intralayer bonds.^12^ Each layer consists of one atomic plane of transition metal atoms, sandwiched between two planes of chalcogen atoms, expressed in general form as an X-M-X structure. This material exhibits semiconducting properties when in the hexagonal crystal coordination, known as the 2H phase.^13,14^

Both QDs and NSs exhibit amplified sensitivity to the immediate chemical and electrostatic environment. This is due to a high surface-area-to-volume ratio and reduced electronic screening.^15,16^ As such, when combined to form mixed dimensional heterostructures, interactions across the junction between the two materials can be harnessed and exploited for enhanced optoelectronic devices.^17−19^ When compared to other low dimensional materials, 2D semiconducting NS exhibit high carrier mobility and a large surface area available for modification, but low optical absorption and PL quantum yield except in the monolayer limit.^20−23^ Conversely, QDs, due to their defined orbital energy levels resulting from quantum confinement in all three spatial dimensions, exhibit a far higher degree of optical absorption and PL quantum yield.^24^ The desirable effects of both can be combined via the rational construction of these heterostructures toward efficient light harvesting or photosensing modalities.^17−19^ QD-NS hybrids have been shown to exhibit efficient PL quenching effects when in close proximity.^5,25−27^ This has been shown to proceed via either direct charge transfer or nonradiative dipole–dipole mediated energy transfer. Though the electronic coupling in such structures has been demonstrated,^28,29^ a key parameter toward the rational construction of these hybrids is to control their distance with nanoscale precision.^30^

Herein we report a strategy to control the assembly of 2D–0D mixed-dimensional heterostructures and tune their electronic coupling employing DNA as a linker. DNA linkers have previously been used for the controlled assembly of MoS_2_ NSs.^31^ Here, we used three different MoS_2_ chemical functionalization strategies (thiol, maleimide, and aryl diazonium) in order to modify the nanosheets with biotinylated DNA strands. This allowed us to then tether streptavidinated QDs to the DNA functionalized MoS_2_ surface via biotin–avidin recognition. The resultant nanohybrid consist of QDs immobilized to the surface of an MoS_2_ nanosheets via DNA as a linker. Nanoscale control over the separation between QDs and NSs was achieved by varying the number of base pairs (bp) constituting the DNA linker. The number of base pairs was varied between 10, 20, and 30 bp, corresponding to separations of 3.4, 6.8, and 13.6 nm, respectively; the quenching of the QDs emission in the constructed mixed dimensional 0D–2D system was probed and was observed to progressively scale as a function of DNA linker employed, hence distance between the two nanomoieties in the hybrids.

## Results

### Preparation of MoS_2_ Nanosheets

MoS_2_ NSs were prepared by surfactant assisted liquid phase exfoliation.^32^ This method results in a polydispersion of NSs sizes, stabilized by surfactant in aqueous media. In order to maximize the fraction of few layered samples obtained in this way, cascade centrifugation was employed. By gradually increasing the speed at which NSs are sedimented and removed from the aqueous surfactant solution, the general dimensions of the obtained NSs can be reduced. Adsorbed surfactant was removed by introducing a three step centrifugal washing process [see the Supporting Information (SI)].

**Figure 1:**
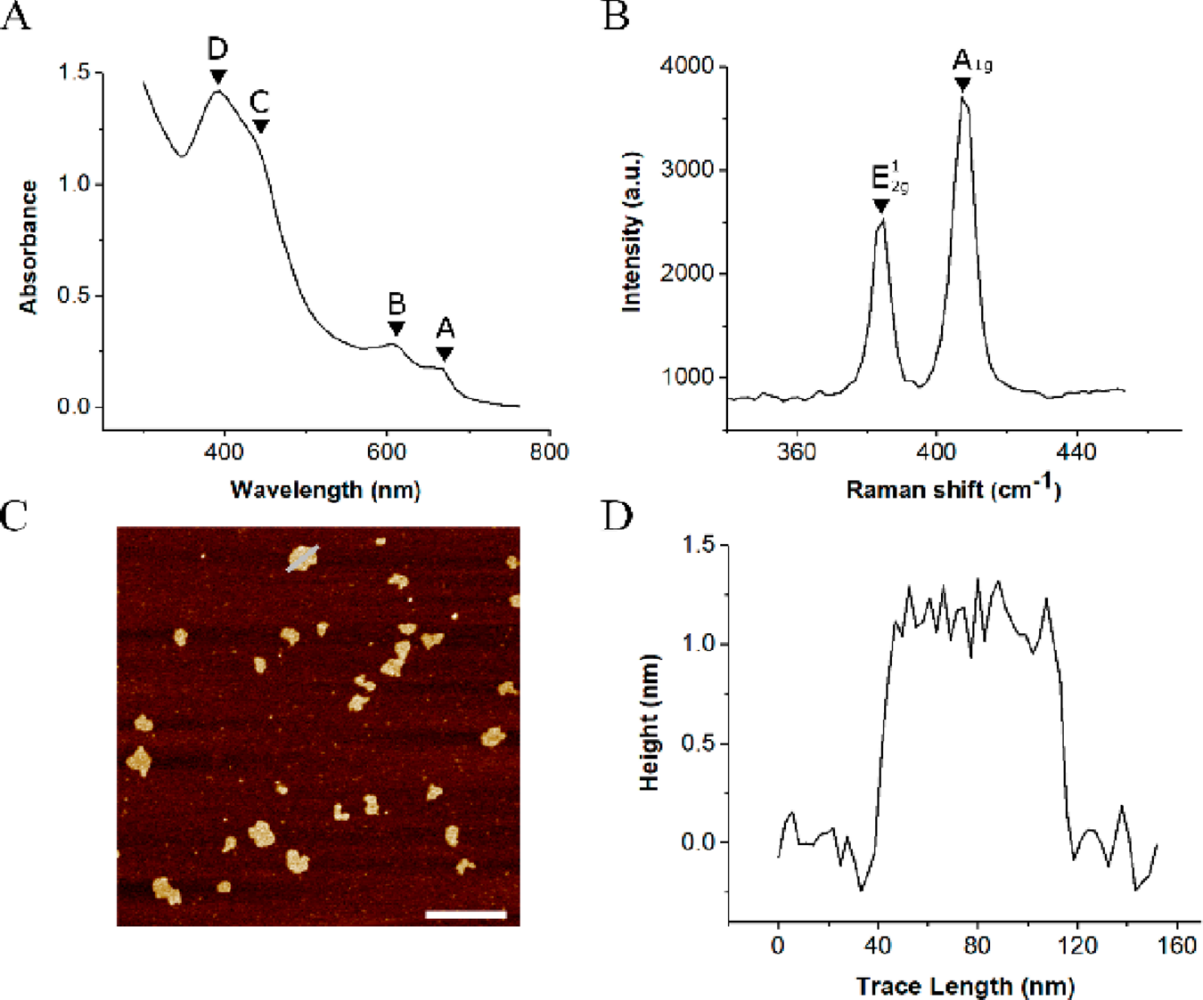
(A) UV–vis spectrum showing the characteristic excitonic peaks of 2H-MoS_2_ nanosheets, confirming their successful exfoliation from bulk. (B) Raman spectrum showing the E^1^_2g_ and the A_1g_ peaks, corresponding to the in- and out-of plane-vibrational modes, respectively. (C)–(D) AFM analysis of exfoliated MoS_2_ nanosheets; extracted height profiles suggest an average lateral size of 44 nm ±13 and an average height of 1.6 nm ±0.3, corresponding to approximately 2–3 layers per NS; the scale bar is equal to 220 nm.

The successful exfoliation of MoS_2_ NSs in the 2H polytype was confirmed by UV–vis and Raman spectroscopy. The characteristic UV–vis absorption spectrum of MoS_2_ NSs in the 2H polytype (Figure 1A) confirms their successful exfoliation from bulk powder. The labeled A and B excitonic peaks correspond to optical transitions across the direct band gap, at the K point of the Brillouin zone. The C and D excitonic peaks have been assigned to transitions at the M point of the Brillouin zone.^13,33^ The presence of these peaks evidence successful exfoliation of few layered samples in the desired polytype. This is further confirmed by the obtained Raman spectrum (Figure 1B). Two clear peaks can be seen at 384 and 405 cm^–1^, corresponding to the in-plane vibrational mode (E^1^_2g_) and the out-of-plane vibrational mode (A_1g_), respectively. When compared to the Raman spectrum of bulk MoS_2_, the shifting of the E^1^_2g_ toward higher wavenumbers and the shifting of the A_1g_ toward lower wavenumbers indicates the presence of few layered samples.^34^ The difference in wavenumber between these vibrational modes can be used to estimate the number of layers exhibited by the nanosheets;^35^ in this case the shift suggests that our nanosheets are composed of two to three layers of MoS_2_ per nanosheet.

Successful size selection was confirmed by atomic force microscopy (AFM). Aqueous solutions of exfoliated NSs were drop cast on muscovite mica for analysis of their dimensions. Analysis of the topographical scan and the representative NS height profile (Figure 1C–D) show that the average diameter of the obtained NS was (44 ± 13) nm and that the average height was (1.6 ± 0.3) nm. Considering an interlayer spacing of 0.65 nm, the measured height corresponds to approximately 2–3 layers per NS. This is in good agreement with the dimension estimates obtained from both the UV–vis spectra^36^ (see the SI and Figure S1) and the Raman spectra.

### DNA Functionalization of Nanosheets

In this study, DNA was chosen as a linker for the subsequent tethering of QDs to MoS_2_ NSs (See Table S1, and the SI for full DNA sequences used). The functionalization of NSs with biotinylated double-stranded DNA (dsDNA) was achieved via three different attachment chemistries, namely, thiol, maleimide, and aryl diazonium salt (Figures S2–S4). This was done in order to facilitate the conjugation of streptavidinated QDs to the NSs surface via a dsDNA linker. The successful surface modification of MoS_2_ NSs with biotinylated dsDNA was confirmed by FTIR and Raman spectroscopy.

Thiols, maleimides, and diazonium salts are well established chemistries employed for macromolecule construction and surface modification.^37−39^ They have been demonstrated to successfully functionalize otherwise pristine and dangling bond free MoS_2_ NSs,^40−43^ toward creating reactive sites for further conjugation.

The thiol-based attachment proceeded via the overnight incubation of MoS_2_ NS with thiol modified single-strand DNA (ssDNA) in aqueous media and at room temperature. Due to the high affinity exhibited by thiols to MoS_2_ nanosheets, these mild reaction conditions were enough to promote attachment.^44^ Unreacted ssDNA was then removed via a centrifugal washing step. This was followed by hybridization of NS-attached ssDNA to a biotin modified complementary strand, resulting in dsDNA attached to the surface of MoS_2_ NSs, exhibiting a biotin modification, toward the subsequent attachment of streptavidinated QDs.

**Figure 2:**
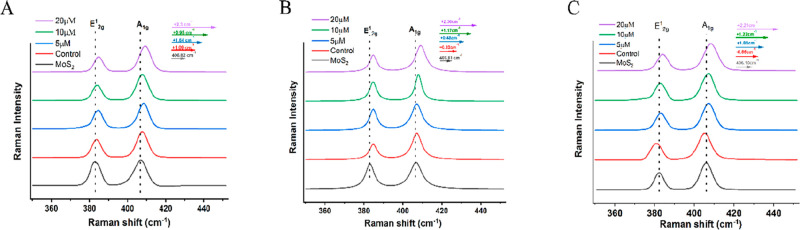
Raman spectra: a shift due to DNA functionalization of MoS_2_ NSs via (A) thiol chemistry, (B) maleimide chemistry, and (C) aryl diazonium salt chemistry. In all three cases the peak wavenumber of the out-of-plane vibrational mode (A_1g_) is monitored as the concentration of functional group modified DNA is increased during attachment to MoS_2_ NSs.

The successful attachment of thiol modified dsDNA to MoS_2_ NSs was confirmed via Raman and FTIR spectroscopy. Notably, NS surface modifications have been shown to alter the frequency of phonon vibrational modes.^45,46^ In order to confirm the chemical modification of MoS_2_ NSs via thiol modified dsDNA, the Raman shift of the A_1g_ out-of-plane vibrational mode was monitored as the concentration of thiol-dsDNA used in the functionalization reaction was increased (Figure 2A), using the Raman response of pristine MoS_2_ as a reference (Figure 2A, black trace). As the concentration of the DNA linker is increased, the A_1g_ vibrational mode is red-shifted toward higher wavenumbers; this is attributed to the successful attachment of thiol groups to the NS surface. The deprotonation of the thiol into a thiolate, followed by the substitution of a sulfur vacancy in the MoS_2_ lattice, has been shown to cause a p-type doping effect and a red shift of the A_1g_ mode.^47^ FTIR spectroscopy performed before and after surface modification (Figure S5) suggests the successful attachment of thiol modified DNA to the NS surface. The weak -SH bending mode^48^ observed at 2545 cm^–1^ is formed only after removal of the protecting group on thiol modified DNA. This mode is then completely damped after functionalization of MoS_2_ NSs, suggesting the deprotonation of the thiol into a thiolate and attachment to the NS surface.

The same methodology as stated above was followed for the other two attachment methods, namely, maleimide and aryl diazonium chemistries. In both cases, an increase in concentration of functional-group-modified dsDNA in the functionalization reaction led to a red shift of the A_1g_ vibrational mode (Figure 2B,C). Unlike for the thiol mediated method, these do not proceed via substitution of sulfur vacancies in the MoS_2_ lattice, but have been shown to form covalent bonds with basal plane sulfur atoms.^42,43^ Both the maleimide and aryl diazonium methods proceed as follows: amine terminated ssDNA is modified with the desired functional group, followed by attachment to the NS surface. It has been shown that chemical modification of the MoS_2_ NS surface with aromatic molecules leads to a red shift of the A_1g_ vibrational mode.^49^ Due to the step employed to remove any unreacted DNA, the redshift in the A_1g_ vibrational mode suggests the successful chemical functionalization of MoS_2_ nanosheets via both maleimide and aryl diazonium chemistries. FTIR spectroscopy was performed to monitor the formation of S–C covalent bonds due to NS functionalization; this was done for both maleimide and aryl diazonium chemistries (Figure S5). Both methods show the formation of an extra peak in the 652 cm^–1^ region after NS functionalization, which has been attributed to the S–C covalent bond.^42,43,48^

### 0D–2D Hybrid Construction

**Figure 3:**
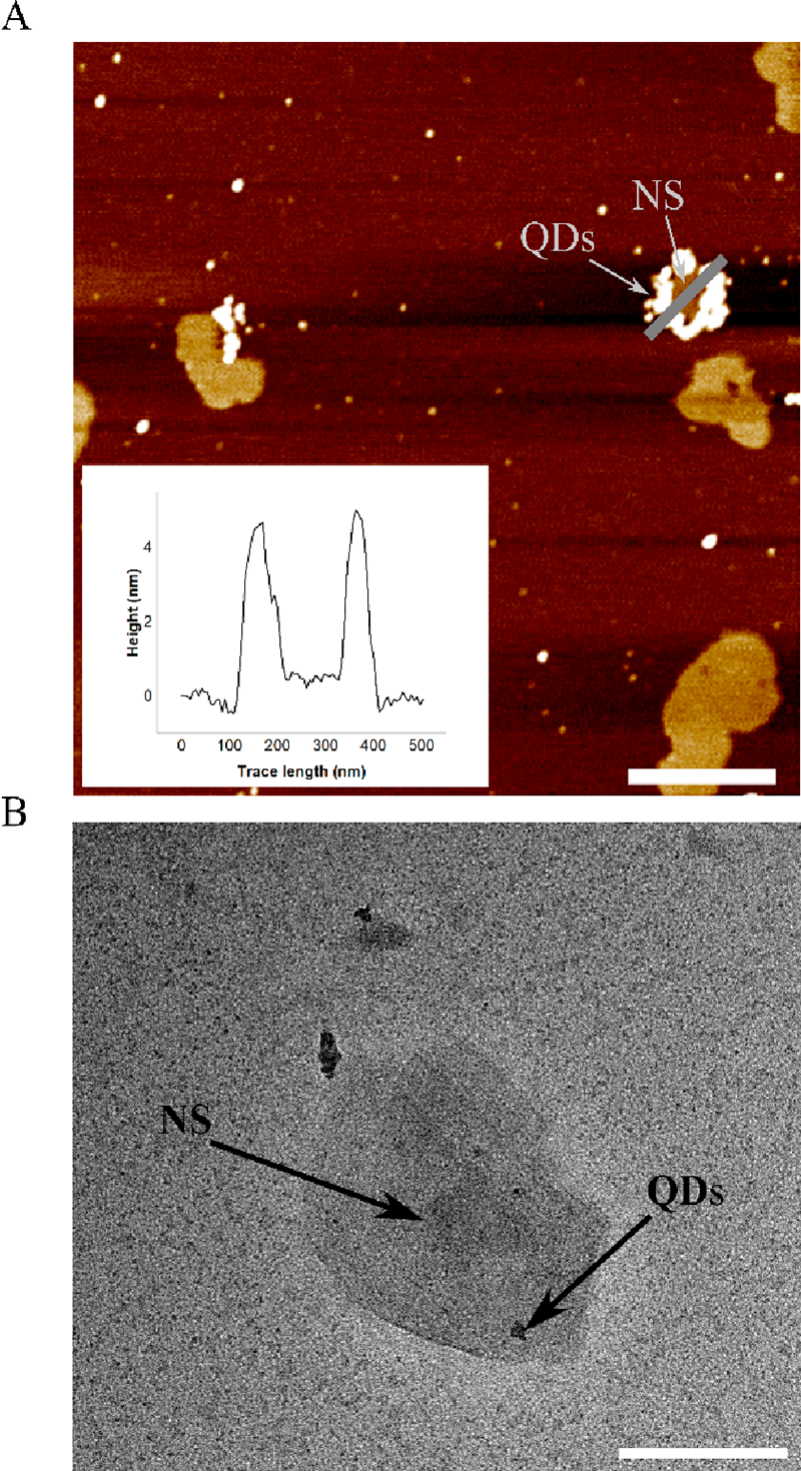
(A) Representative AFM topographical image showing an MoS_2_ NS conjugated to a cluster of QDs via maleimide modified DNA, scale bar = 520 nm. (B) TEM representative image of QDs conjugated to a single MoS_2_ NS. The concentration of QDs used was reduced in order to resolve single QDs conjugated to MoS_2_ NSs, scale bar = 200 nm.

Once the successful attachment of dsDNA to MoS_2_ nanosheets was achieved, these hybrids were used as the building blocks for the construction of MoS_2_-DNA-QD heterostructures. The MoS_2_-DNA conjugates were incubated with streptavidinated CdSe/ZnS core–shell QDs in order to tether the nanocrystals to the DNA-modified NS via biotin–avidin recognition (see the SI, Materials and Methods). Briefly, this was achieved via a simple overnight incubation of colloidal streptavidinated QD solution with the dsDNA functionalized MoS_2_ nanosheets. The successful conjugation of QDs to MoS_2_ NSs, using DNA as a linker, was confirmed by AFM and transmission electron microscopy (TEM). A representative AFM images of the hybrids is shown in Figure 3A; the inset shows a cross sectional height profile showing features measuring approximately 4 nm clustered around a few-layered NS. Control experiments were carried out whereby MoS_2_ NSs and streptavidinated QDs where mixed without the DNA linker (Figure S6), confirming that the clustering of QDs around NSs was due to the employed functionalization strategies. TEM analysis of dilute solutions of QD conjugated MoS_2_ nanosheets, cast on copper grids, show individual QDs on the surface of MoS_2_ NSs (Figure 3B). Pristine colloidal QD solution was imaged via TEM (Supporting Information, Figure S7), confirming that the features observed in the functionalized sample are single and small clusters of QDSs. Both the AFM and TEM analyses confirm the successful tethering of streptavidinated QDs to MoS_2_ NSs.

### Photoluminescence Quenching in the Heterostructures

In order to tune the electronic coupling between QDs and MoS_2_ NSs, the length of the DNA linker between them was varied. This was achieved by using dsDNA strands of 10, 20, and 30 base pair (bp) length, equating to a separation of 3.4, 6.8, and 13.6 nm, respectively. We monitored the steady state photoluminescence (PL) emission of the MoS_2_-tethered QDs as a function of the length of the DNA linker by which they were attached.

**Figure 4:**
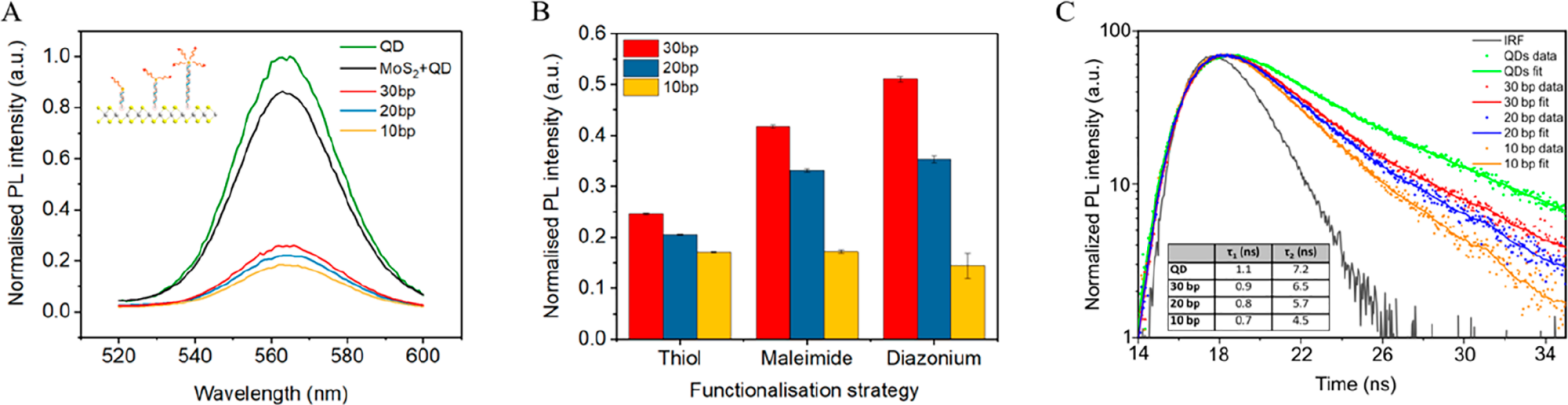
(A) Steady-state photoluminescence spectra of pristine QDs (green) and each of MoS_2_ hybrids (using thiol modified dsDNA) as the distance between QDs and NSs is decreased, and control experiment (black) of mixed MoS_2_ NSs and QDs with no linkers; the inset shows a schematic representation of DNA linked hybrids. (B) Histogram showing peak QD emission intensity for a decrease in distance between QDs and NSs for the three attachment chemistries used, namely, thiol, maleimide, and aryl diazonium. (C) Time resolved photoluminescence of QD emission, taken as the distance between MoS_2_ NSs and QDs is controllably varied via a DNA linker, attached via thiol chemistry; the inset table shows the PL lifetimes for each sample.

The PL spectrum of QDs as the separation between QDs and MoS_2_ nanosheets was varied using thiol-anchored dsDNA shows a decrease of the PL intensity of the nanocrystals as the separation between QD and NS is decreased (Figure 4A). Using the PL response of pure QD solution as a reference, the 30 bp sample displayed a reduction of the QDs PL of 75%, the 20 bp of 79%, and the 10 bp sample of 82%. This confirms the distance dependent electronic coupling between MoS_2_ NSs and QDs. PL spectra for MoS_2_-DNA-QD hybrid construction via the maleimide and aryl diazonium methods also confirmed the successful distance-dependent electronic coupling of NSs and QDs (Figure S8).

The emission of the QDs in the hybrids as monitored via PL as the dsDNA linker is sequentially shortened for the three attachment chemistries; namely, thiol, maleimide, and aryl diazonium show an increase in quenching efficiency as separation is decreased in all cases (Figure 4B). This suggests both efficient electronic coupling between QDs and MoS_2_ NSs and successful construction of mixed-dimensional hybrids between NSs and QDs using dsDNA as a linker to control their distance with nanoscale accuracy.

To further investigate the electronic coupling mechanism responsible for the quenching of QDs emission, we examined the radiative lifetime of the thiol-based QDs-MoS_2_ hybrids by means of time-resolved photoluminescence measurement (TRPL) (Figure 4C). The emission of QD solution shows a characteristic biexponential decay behavior.^50^ The shorter lifetime (τ_1_) is commonly attributed to the recombination of initially populated core states, while the longer one (τ_2_) to radiative recombination of excitons involving surface states. No significative difference in the PL kinetic was observed by simply mixing the QDs and MoS_2_ sheets in the absence of DNA linkers. Conversely, in the QD hybrids formed with MoS_2_ through DNA linkage, both the τ_1_ and τ_2_ components of the QDs PL were observed to progressively shorten as the QDs approached the MoS_2_, while preserving the biexponential decay (see Figure 4c inset table). As previously pointed out,^51^ this overall lifetime reduction is indicative of energy transfer (ET) of the excitons into MoS_2_, which is competitive with the present relaxation pathways (surface trap and exciton recombination) and may be effective even at long-range (few nanometers distance). Adding an additional ET decay channel increases the overall exciton recombination rate and decreases the overall PL lifetime. The distance dependence behavior of TRPL as the dsDNA linker is sequentially shortened shows a decrease of both lifetimes. This agrees with the inverse distance dependence expected for the rate of ET, and with recent observation made on similar QD-MoS_2_ heterostructures.^25^

## Conclusions

In summary, the assembly and characterization of 0D–2D mixed-dimensional heterostructures was achieved linking MoS_2_ nanosheets and CdSe/ZnS core–shell QDs with DNA as a molecular ruler, employing three different attachment chemistries, namely, thiol, maleimide, and aryl diazonium. The successful attachment of dsDNA linkers via these three functionalization chemistries was confirmed by Raman and FTIR spectroscopy. PL measurements demonstrated that this allowed us to tune the electronic coupling between NSs and QDs via precise control over the separation between them, achieved by varying the bp length of the DNA linker used. This strategy is of general applicability for the construction of DNA-linked mixed-dimensional heterostructures with nanoscale control over the separation between units, allowing for modulation of their electronic coupling.

## Supporting Information Available

Additional experimental details, materials and methods, control experiments and schematics of chemical functionalization strategies (PDF)
