# Supplementary material for: Nanoscale Control of DNA-Linked MoS2-Quantum Dot Heterostructures
Source: Bioconjug Chem. 2022 Aug 15;34(1):78–84. doi: 10.1021/acs.bioconjchem.2c00285 (PMC9853502; doi:10.1021/acs.bioconjchem.2c00285)
Supplement: Supplementary file 1 — bc2c00285_si_001.pdf [file bc2c00285_si_001.pdf]

Supporting information

Nanoscale control of DNA-linked MoS_2-Quantum Dot Heterostructures

Teymour Talha-Dean[†,§,‡], Kai Chen[ǀ,‡], , Giulia Mastroianni[ɸ], Felice Gesuele[Γ] , Jan Mol[†,*], Matteo Palma[ǀ,*]

[†] Department of Physics and Astronomy, Queen Mary University of London, London, E1 4NS, United Kingdom

[ǀ] Department of Chemistry, Queen Mary University of London, London, E1 4NS, United Kingdom

[§] Institute of Materials Research and Engineering (IMRE), Agency for Science, Technology and Research (A*STAR), 138634, Singapore

[ɸ] School of Biological and Behavioral Sciences, Queen Mary University of London, London, E1 4NS, United Kingdom

[Γ] Department of Physics "Ettore Pancini", University of Naples "Federico II", Via Cinthia, 21 Ed. 6, 80126 Napoli, Italy

*E-mail: j.mol@qmul.ac.uk,

*E-mail: m.palma@qmul.ac.uk

Materials and methods

Materials

All the DNA oligonucleotides are ordered from IDT, and the details of these sequences are presented in Table S1. Molybdenum disulfide (MoS_2), sodium cholate, Tris (2-carboxyethyl) phosphine (TCEP), N-Succinimidyl 3-maleimidobenzoate, 95%, 4-Nitrobenzenediazonium tetrafluoroborate, Tin Chloride ($SnCl_2$) are ordered from Sigma-Aldrich. The solvents used in this work including acetonitrile (ACN) ethanol and acetone are analytical agent. Water used in this work is Milli-Q water.

Table S1. Sequences of DNA

Label	Sequence
Amine-10bp	5'-Amine- CAG GCT CAG G-3'
Amine-20bp	5'-Amine- TGC TAT GCA GGC TCA GG-3'
Amine-30bp	5'-Amine- TGC TAT GCA GCG GTC AAC TAC AGG CTC AGG-3'
Thiol-10bp	5'-Thiol- CAG GCT CAG G
Thiol-20bp	5'-Thiol- TGC TAT GCA GCA GGC TCA GG
Thiol-30bp	5'-Thiol- TGC TAT GCA GCG GTC AAC TAC AGG CTC AGG
Biotin-10bp	5'-Biotin- CCT GAG CCTG-3'
Biotin-20bp	5'-Biotin- CCT GAG CCT GCT GCA TAG CA-3'
Biotin-30bp	5'-Botin- CCT GAG CCT GTA GTT GAC CGC TGC ATA GCA-3'

Preparation and characterization of MoS_2 nanosheet

In this project, liquid phase exfoliation (LPE) is used for preparing high quality MoS_2 nanosheet. Then cascade centrifugation method is introduced to select and separate MoS_2 nanosheet with proper size. The information of lateral size and number of layers (thickness) can be obtained by measuring its UV-Vis spectrum and determined by the equations below. And then relevant results can be confirmed by Atomic Force Microscope (AFM) directly.

$$L\ (\mu m) = \frac{3.5\frac{Ext_B}{Ext_{345}} - 0.14}{11.5 - \frac{Ext_B}{Ext_{345}}}$$

Where L is the length of the MoS_2 nanosheet, Ext_B is the absorbance of exciton B, Ext_{345} is the absorbance at 345nm in the UV-Vis spectrum.

$$N_{MoS2} = 2.3 \times 10^{36} e^{\frac{-54888}{\lambda A}}$$

Where N is the number of layers of MoS_2 nanosheet, λ_A is the position of exciton A.

The vibrational property of MoS_2 nanosheet is also distinct than their bulk counterpart. There are four Raman-active modes, and one IR-active mode can be observed in the unit cell of the bulk MoS_2 crystal as viewed along the [1000] direction. In Raman spectrum, which is a strong characterization for measuring the vibrational mode, the signals for E^1_{2g} and A_{1g} mode can be obtained clearly. And the gap between these two signals is highly dependent on the thickness of MoS_2 nanosheet. So, it could also be a strong method to characterize the number of layers for MoS_2 nanosheet.

The detail of preparation is concluded as follow. At first, bulk MoS_2 powder (7mg) and sodium cholate (3mg) are added into a glass tube (3mL) then dissolved with 1mL water. The mixture is then sonicated overnight (Branson Ultrasonics™ CPXH Series Ultrasonic Cleaning Bath, 110W). After sonication, the mixture is divided to two tubes (500µL each) for the cascade centrifugation next. The centrifugation starts under 2000 rpm for 45 mins, and the sediment is discarded for removing the unexfoliated MoS_2. Then the supernatant is subjected to further centrifugation with higher speed (3000 rpm) for 45 mins and the nanosheet can be collected from the sediment. By repeating this procedure, different size of MoS_2 nanosheets are obtained from the sediment while the supernatant subjecting higher centrifugation speed (6000 rpm, 8000rpm, 12000rpm and 15000rpm). In this work, the nanosheets which obtained from the sediment of 8000rpm are selected for further functionalization.

Functionalization of MoS_2 nanosheet

MoS_2 nanosheet which prepared as the procedure aforementioned is functionalized by three strategies based on DNA in this work.

Fig. S1 shows the functionalization route of maleimide strategy. 10µL N-Succinimidyl 3-maleimidobenzoate (3mM) and 10 µL amine DNA are dissolved in 10 µL ACN and 30 µL buffer (pH 7.2) for 3 hours. Then the sample, sodium acetate and ethanol (1:0.1:2.5, V/V/V) are mixed and bathed with ice for 30mins. The white sediment will be collected by centrifugation (15000 rpm, 10mins). Products from last step will be redispersed in 50 µL ACN solution, and 50 µL MoS_2 nanosheet and 0.5 µL triethylamine are added in sequence. After 12 hours incubation, mixture will be purified by centrifugation (12000 rpm). The black sediment is redispersed in 90 µL DPBS buffer, then 10 µL complementary DNA (biotin modified) will be added. Finally, the QD 565 (CdSe/ZnS, streptavidin modified) will be attached on the double strand DNA.

Fig. S2 is the schematic of diazonium salt route. At first, MoS_2 nanosheet is functionalized by 4-nitrobenzene tetrafluoroborate (4-NBD) at 35°C for 1 hour. The reduction procedure of Nitro is performed by mixing sediment from last step with 50 µL $SnCl_2$ (0.2M) and 50 µL HCl (0.2M) for 24 hours. Then products are purified by centrifugation (12000rpm) and the sediment is collected. Next, diazotization is executed by dissolving sediment in 10 µL specific solution (6% $NaNO_2$ in 1M HCl, V/V) for 30 mins. After centrifugation, sediment is dissolved in 100 µL DPBS buffer and then 10 µL complementary DNA (biotin modified) is added. Finally, QD 565 is conjugated on to double strand DNA.

Thiol strategy can be achieved by mixing 1 µL (100 µM) thiol modified DNA with 100 µL MoS_2 nanosheet overnight directly Fig. S3. The protection group of thiol DNA which used here was removed in advance. The remove of protection group was performed by mixing 1µL

TCEP (10mM) and 1 µL (100 µM) thiol modified DNA together for 3 hours. After thiol DNA attached on MoS_2 nanosheet, free DNA is removed by centrifugation. And the sediment is redispersed into 100 µL DPBS buffer. 1 µL complementary DNA (100 µM) which is modified by biotin group is added and hybridized afterwards. Then QD 565 is conjugated on to double strand DNA.

Characterization

Steady-state Photoluminescence (PL) spectroscopy was performed by Agilent Cary PL spectrometer. All the samples for PL characterization were prepared in water solution (Mili Q water) with a total volume of 50 µL. And the length path of the quartz cell is 1 cm. The general parameters for PL characterization are presented as follow: 1) excitation wavelength is 420 nm. 2) excitation slit and emission slit values/settings are 10 nm. 3) PMT voltage is high mode.

Ultraviolet-Visible spectroscopy (UV-Vis) was collected by Shimadzu UV-3600 uv-vis spectrometer. All the samples are prepared in water solution with a total volume of 50 µL. And the length path of the quartz cell is 1 cm.

Time resolved PL measurements (TRPL) were performed for the QDs and Hybrid solution. We employed a 450nm, 2 ns excitation laser source. The PL was collected by means of an optical lens system and spectrally selected, by means of band pass filter, in the 500-600 nm range. It was sent to a fast 1.6GHz avalanche photodiode to visualize (directly on a 1GHz oscilloscope) the decay kinetics.

Fourier Transform Infrared Spectroscopy (FTIR) was performed by PerkinElmer spectrum 65 FTIR spectrometer.

Raman spectroscopy was collected by Renishaw inVia Reflex Spectrometer System for Raman StreamLineTM spectral analysis and rapid imaging system. Three laser sources are

equipped which are 442 nm, 633 nm and 785 nm. 442 nm was selected as the excitation source in the collection of Raman spectrum in this work.

Atomic Force Microscopy (AFM) was carried out by Bruker Dimension Icon in PeakForce Tapping mode with ScanAsyst Air tips from Bruker. The solution was cast on discs of muscovite mica (Agar Scientific) which was treated by $MgCl_2$ before loading. Subsiquenlty it was subjected to a wash process and then blown dry with compressed air.

Transmission Electron Microscopy (TEM) were performed by JEOL JEM2100 Plus which operated at 200KV and OneView camera (Gatan). A Gatan model 626 cryoholder sitting, inserted a-top its model 655 vacuum pumping station.

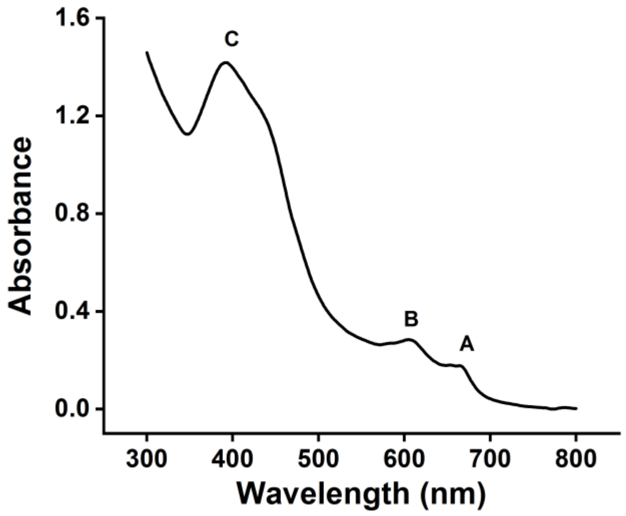

Fig. S1 Uv-Vis spectrum for the MoS_2 nanosheet after selection process

According to the equations in experimental section, the lateral size of MoS_2 nanosheet used in this work can be calculated based on Fig. S1, and the details presented as follow:

$$L\ (\mu m) = \frac{3.5\frac{0.28408}{1.12624} - 0.14}{11.5 - \frac{0.28408}{1.12624}} = 0.066042 \qquad N_{MoS2} = 2.3 \times 10^{36} e^{\frac{-54888}{664}} = 2.896$$

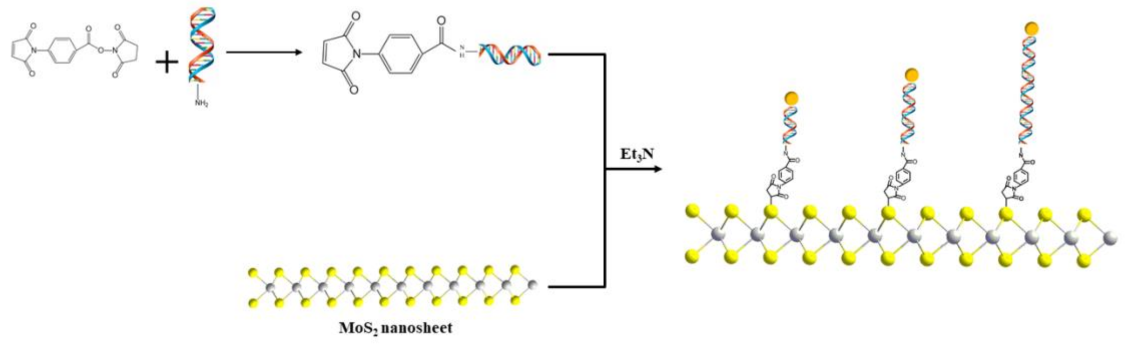

Fig. S2 Schematic of maleimide strategy

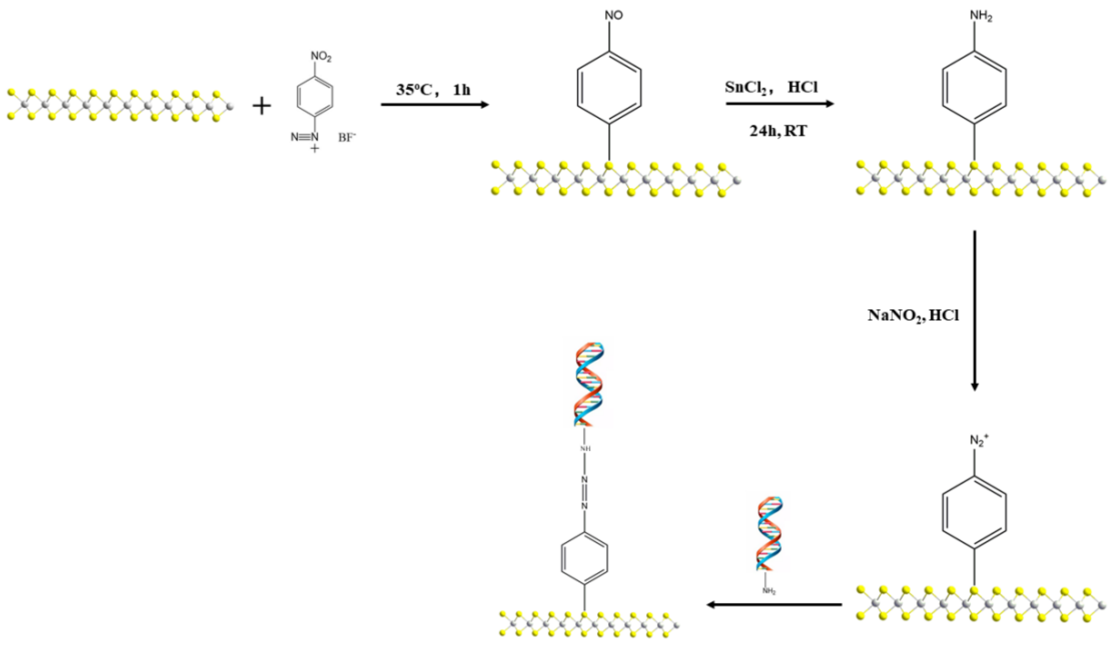

Fig. S3 Schematic of Diazonium salt functionalization strategy

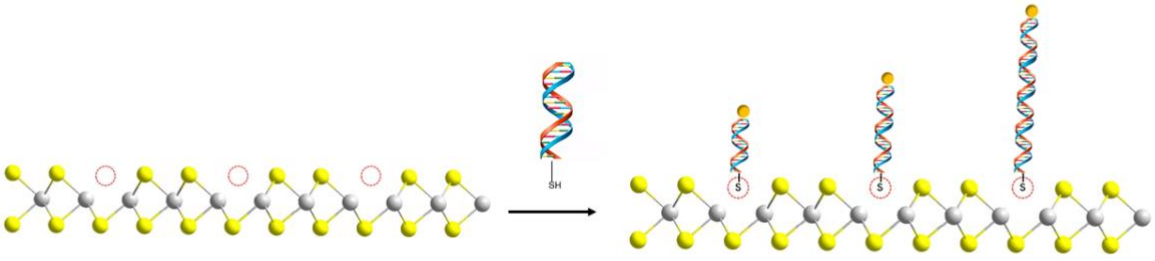

Fig. S4 Schematic of thiol strategy

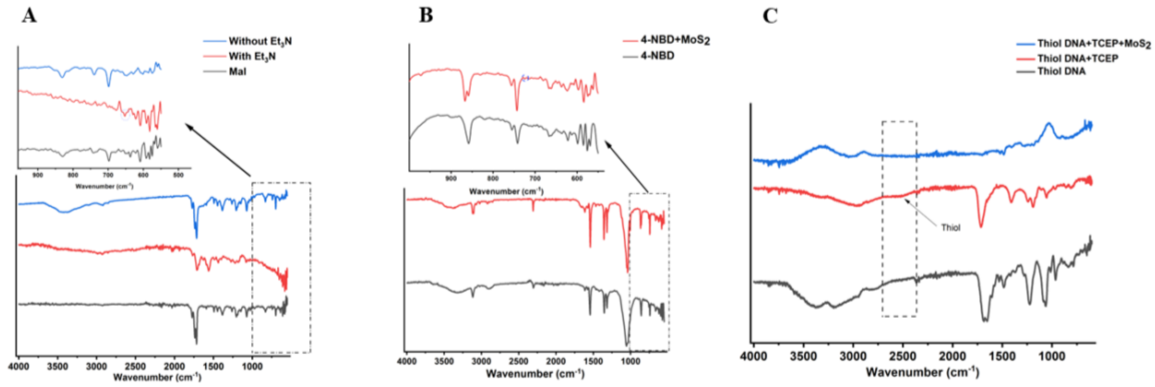

Fig. S5 FTIR spectrum for: (A) hybrids functionlized by Maleimide strategy; (B) hybrids functionalized by diazonium salt strategy; (C) hybrids functionized by thiol strategy

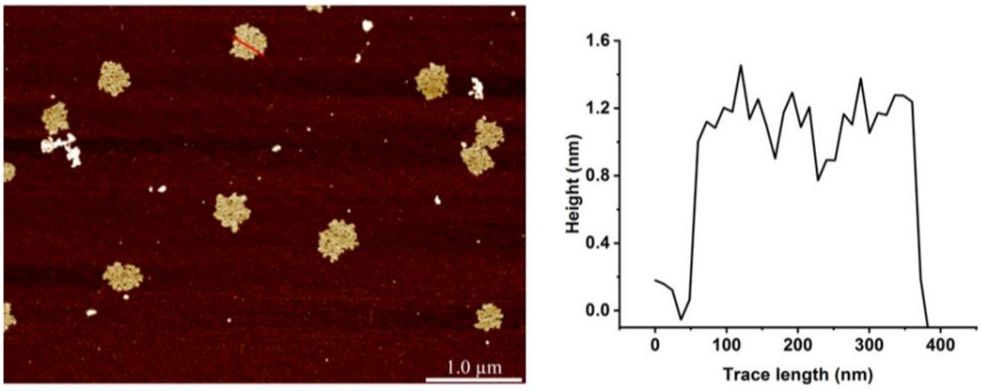

Fig. S6 AFM image for physical mixing of MoS_2 nanosheets and QDs: no QDS on MoS_2 NSs are presnet

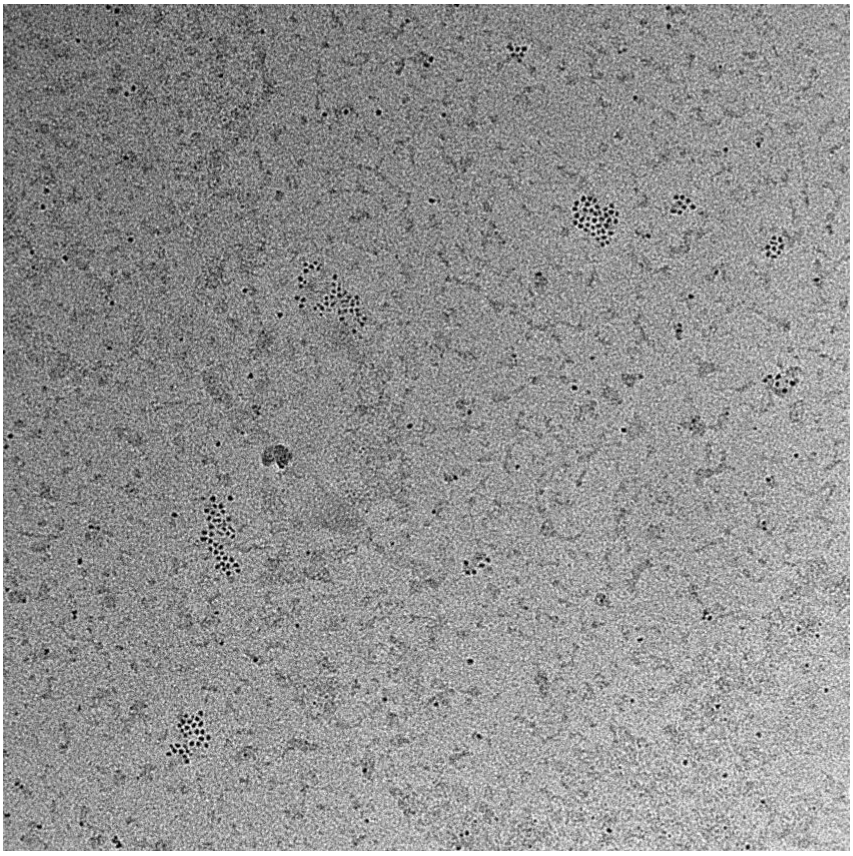

Fig. S7 TEM image for pristine QDs (CdSe/ZnS)

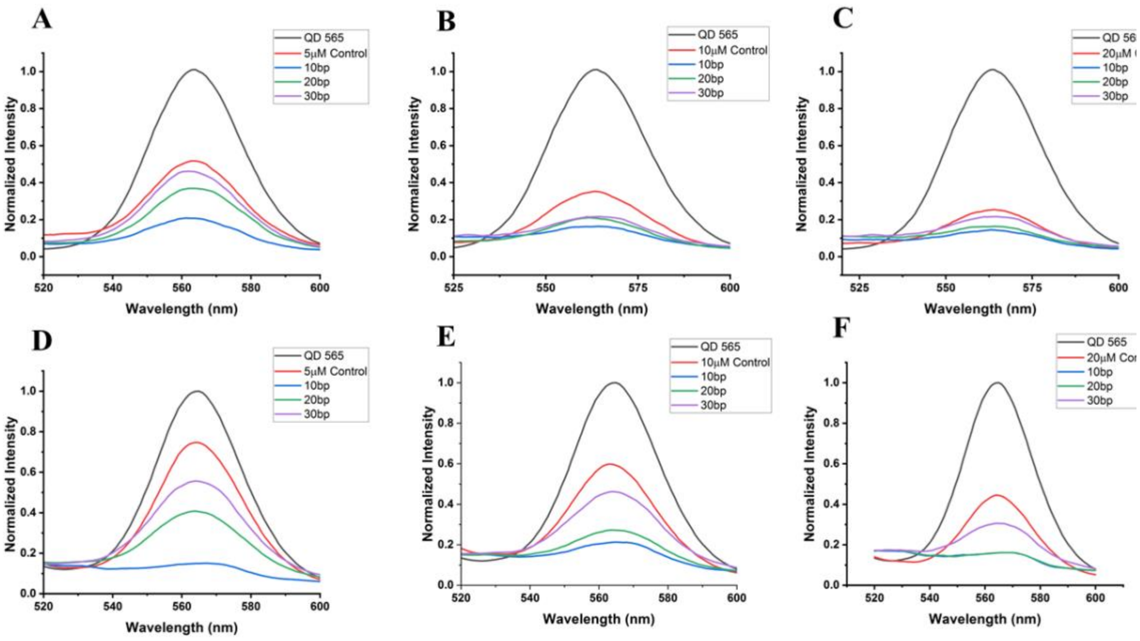

Fig. S8 PL spectrum for quenching behaviour of: (A-C) Maleimide functionalized hybrid under 5 μM, 10μM and 20μM DNA linker applied; (D-F) Diazonium salt functionalized hybrid under 5 μM, 10μM and 20μM DNA linker applied.
